# MRI-Based Pancreatic Atrophy Is Associated With Malignancy or Invasive Carcinoma in Intraductal Papillary Mucinous Neoplasm

**DOI:** 10.3389/fonc.2022.894023

**Published:** 2022-06-03

**Authors:** Tingting Lin, Xin Chen, Jingjing Liu, Yingying Cao, Wenjing Cui, Zhongqiu Wang, Cheng Wang, Xiao Chen

**Affiliations:** ^1^ Department of Radiology, Affiliated Hospital of Nanjing University of Chinese Medicine, Nanjing, China; ^2^ Department of Radiology, Shanghai Sixth People’s Hospital, Shanghai, China; ^3^ Department of Radiology, Nanjing Drum Tower Hospital, Nanjing, China; ^4^ Institute of Radiation Medicine, Fudan University, Shanghai, China

**Keywords:** pancreatic atrophy, intraductal papillary mucinous neoplasms, pancreas, malignancy, invasive carcinoma

## Abstract

**Background:**

Abrupt change in the caliber of the main pancreatic duct (MPD) with distal pancreatic atrophy (PA) was considered as one of worrisome features in the International Association of Pancreatology guideline and American College of Gastroenterology guideline for the management of intraductal papillary mucinous neoplasms (IPMNs). However, this feature was not included in other guidelines. Moreover, the association between PA alone and malignancy in IPMNs has not been fully evaluated. In the present study, we investigated the role of image-based PA in identifying malignant IPMNs or invasive carcinoma.

**Methods:**

A total of 186 patients with IPMNs were included for analysis. The tumor size, location, MPD diameter, presence of a mural nodule (MN), and PA were evaluated using magnetic resonance imaging. Demographic information and serum carbohydrate antigen 19-9 and carcinoembryonic antigen (CEA) levels were also collected. IPMNs with high-grade dysplasia and associated invasive carcinoma were regarded as malignant IPMNs.

**Results:**

PA was observed in 34 cases (18.3%). The occurrence of malignant IPMNs or invasive carcinoma in patients with PA were significantly higher than in those without PA (52.9% vs. 22.3%; 44.1% vs. 8.9%, all P < 0.01). Multivariate logistic regression analysis showed that PA was an independently associated factor for malignant IPMNs [odds ratio (OR) = 2.69, 95% confidence interval (CI): 1.07-6.78] or invasive carcinoma (OR = 7.78, 95%CI: 2.62-23.10) after modified with confounders. Subgroup analysis in MPD-involved IPMNs also indicated that PA was an independently associated factor for invasive carcinoma (OR = 9.72, 95%CI: 2.43-38.88). PA had a similar performance with MPD plus MN [the area under the curve (AUC) was both 0.71] in identifying malignancy. PA had a higher performance in identifying invasive carcinoma in MPD-involved IPMNs than MN (AUC = 0.71 vs. 0.65, P = 0.02).

**Conclusion:**

Our data showed that imaging-based PA was associated with malignancy or invasive carcinoma regardless of abrupt change in the caliber of MPD in IPMNs. PA had an acceptable performance in identifying malignant IPMNs.

## Introduction

Intraductal papillary mucinous neoplasm (IPMN) of the pancreas is a cystic tumor with papillary growth and mucin secretion in the duct. IPMNs have a great potential of malignant transformation. The management of IPMNs remains a challenge in clinical practice (1) because none of the guidelines is chiefly complete (2). Nowadays, a tumor size ≥3.0 cm, the presence of an enhanced mural nodule (MN), main pancreatic diameter ≥10 mm, thickened/enhancing cyst walls, obstructive jaundice, and elevated carbohydrate antigen 19-9 (CA19-9) levels have been widely used as risk factors to predict malignancy (3). Some models with acceptable performance were also established based on those risk factors (4, 5).

Pancreatic atrophy (PA) is usually occurred in pancreatic ductal adenocarcinoma. Excessive mucin secretion from IPMNs can obstruct the main pancreatic duct (MPD), leading to PA and fibrosis (6). Currently, nine guidelines (2) proposed radiological and clinical criteria to assess the malignant potential of pancreatic IPMNs. Among these, only two guidelines mentioned that PA was a risk factor for malignancy. The International Association of Pancreatology (IAP) guideline (2017) (3) mentioned that abrupt change in the caliber of MPD with distal PA was a worrisome feature of malignant IPMNs, and the American College of Gastroenterology (ACG) clinical guideline (2018) (7) suggested the abrupt change in the caliber of MPD with distal PA as a high-risk characteristic for malignant mucinous pancreatic cysts.

Interestingly, some studies have found that pathological PA was also related to invasive carcinoma or malignant IPMNs (8–11). Saito et al. (8) found that PA and fibrosis were more common in a high grade of IPMNs than that in a low grade (50%-76% vs. 6%). In addition, several studies showed that abrupt MPD caliber change with distal PA reported during radiological examinations was associated with malignant IPMNs or invasive carcinoma in all IPMNs and branch-duct IPMNs (BD-IPMNs) (12–20). By contrast, some studies did not observe such association in univariate or multivariate analysis (21–26). Moreover, whether PA alone (regardless of abrupt MPD caliber change) is associated with malignant IPMNs is still not fully understood. To the best of our knowledge, only one study with a small sample size (n = 55) showed that PA is associated with malignant IPMNs in univariate analysis (13). However, whether PA is an independent risk factor was not investigated. Therefore, the main purpose of this study is to investigate whether image-based PA can independently indicate the presence of malignant lesions in IPMNs.

## Materials and Methods

### Patients

The retrospective study was approved by the Ethnic Committee of Affiliated Hospital of Nanjing University of Chinese Medicine. A total of 214 patients with pathologically proved IPMNs were found in our institutions during January 2016 to May 2021. Among these patients, 29 patients were excluded for the following reasons: the absence of magnetic resonance imaging (MRI) or MRI examinations at an outside hospital (n = 16); absence of contrast-enhanced examinations (n = 5); and other pancreatic tumors (n = 8). Because MRI or MR cholangiopancreatography (MRCP) has an advantage in identifying BD-IPMNs, we only included patients with MRI examinations. The clinical information, including demographic data (age, gender); tumor biomarkers [CA19-9 and carcinoembryonic antigen (CEA)]; and medical history of diabetes mellitus (DM), pancreatitis, and jaundice, were recorded from the medical system. DM was considered if plasma glucose levels were higher than 7.1 mmol/L or if there is a history of DM.

### Imaging Methods and Imaging Analysis

MR scans were performed by using a 3.0-T or 1.5-T unit (Signa HDx 3.0-T; GE Medical Systems, Milwaukee, WI, United States, or Achieva 1.5-T; Philips, Amsterdam, The Netherlands). Conventional axial, sagittal, and coronal T1-weighted turbo spin-echo imaging sequence (without and with gadolinium), fast spin-echo T2-weighted fat-suppressed sequence (echo time/repetition time [TE/TR]: 4,000–8,000/80–90 ms). MRCP was performed using heavily T2-weighted fast acquisition spin echo sequence (TR/TE: 2,400–6,000/500–800 ms). Contrast enhanced imaging was also performed after the intravenous injection of 0.1 mmol/kg gadolinium (2.5 ml/s).

The following imaging parameters were collected: tumor location (head–neck or body–tail), tumor size, MPD diameter, and the presence of enhanced MN with a size ≥5 mm. The MPD diameter was measured at the point of the maximally dilated pancreatic duct (16). Enhanced MN was considered if there were any enhancing solid papillary protuberances within the lesions (16). PA was considered if the ratio between the MPD diameter and the width of the pancreas parenchyma is larger than 0.5 (27).

### Pathological Examinations

IPMN was divided into three subtypes based on the degree of involvement of the pancreatic ductal system: main duct (MD), BD, and mixed type (MT). The pathological grade of IPMN was classified as low-intermediate dysplasia, high-grade dysplasia, and invasive adenocarcinoma according to the World Health Organization guideline. Those IPMNs with high-grade dysplasia and associated invasive carcinoma were regarded as malignant IPMNs.

### Statistical Analysis

Data management and statistical analysis were all performed by using SPSS 16.0 (IBM, Armonk, NY, United States). CA19-9, CEA, the tumor size, and MPD diameter were divided into two groups: <37.0 and ≥37.0 U/ml; <5.0 and ≥5.0 ng/ml; ≥3.0 and **<**3.0** **cm; and ≥1.0 and **<**1.0** **cm. Two-tailed independent t tests or the Mann–Whitney U-test were adopted to compare the variables between patients with and without PA. A chi-square test or Fisher exact test was used to compare categorical variables. Univariate and multivariate logistic regression analyses were used to investigate the association between the presence of PA and malignant IPMNs or invasive carcinoma. Receiver operating characteristic (ROC) curves were conducted to show the performance of PA alone or combination with other markers in identifying malignant IPMNs or invasive carcinoma. Two-tailed P-values < 0.05 were considered as a statistical significance.

## Results

### Characteristics of Subjects

A total of 186 patients met the selection criteria and were included in the analysis. The characteristics of subjects with PA and without PA are shown in **Table 1**. PA occurred in 34 (18.3%) patients with IPMNs. Factors such as age, sex, CEA level, and DM did not differ between the two groups (all P > 0.05). The prevalence of high CA19-9 levels (>37 U/ml) in patients with PA was significantly higher than those without PA (P < 0.05). Regarding the morphological features of IPMNs, the occurrences of an MPD diameter ≥1.0 cm, cyst size ≥3.0 cm, and MN were more common in patients with PA than those without PA (all P < 0.01), but no such difference was observed in the tumor location (P > 0.05). The incidence of PA was higher in MD-involved IPMNs than that in BD-IPMNs (P < 0.01).

**Table 1:** Characteristic of IPMN patients.

	PA (n = 34)	Non-PA (n = 152)	P
Age	65.3 ± 10.2	62.8 ± 8.9	>0.05
Sex (M/F)	23/11	91/61	>0.05
MPD diameter ≥ 1.0 cm	10	17	<0.01
Size ≥ 3.0 cm	26	63	<0.01
Mural nodule (yes)	10	15	<0.01
Location			>0.05
Head–neck	22	96	
Body–tail	12	56	
IPMN type			<0.01
MD-involved	26	64	
BD-IPMN	8	88	
CA19-9 > 37 (U/ml)	12	25	<0.05
CEA > 5.0 (ng/ml)	8	20	>0.05
Malignancy	18	34	<0.01
Invasive carcinoma	15	13	<0.01
Diabetes	8	24	>0.05

BD, branch duct; CA19-9, carbohydrate antigen 19-9; CEA, carcinoembryonic antigen; MD, main duct; MPD, main pancreatic duct; PA, pancreatic atrophy.

### Occurrences of Malignancy and Invasive Carcinoma in Patients With PA

**Figure 1:**
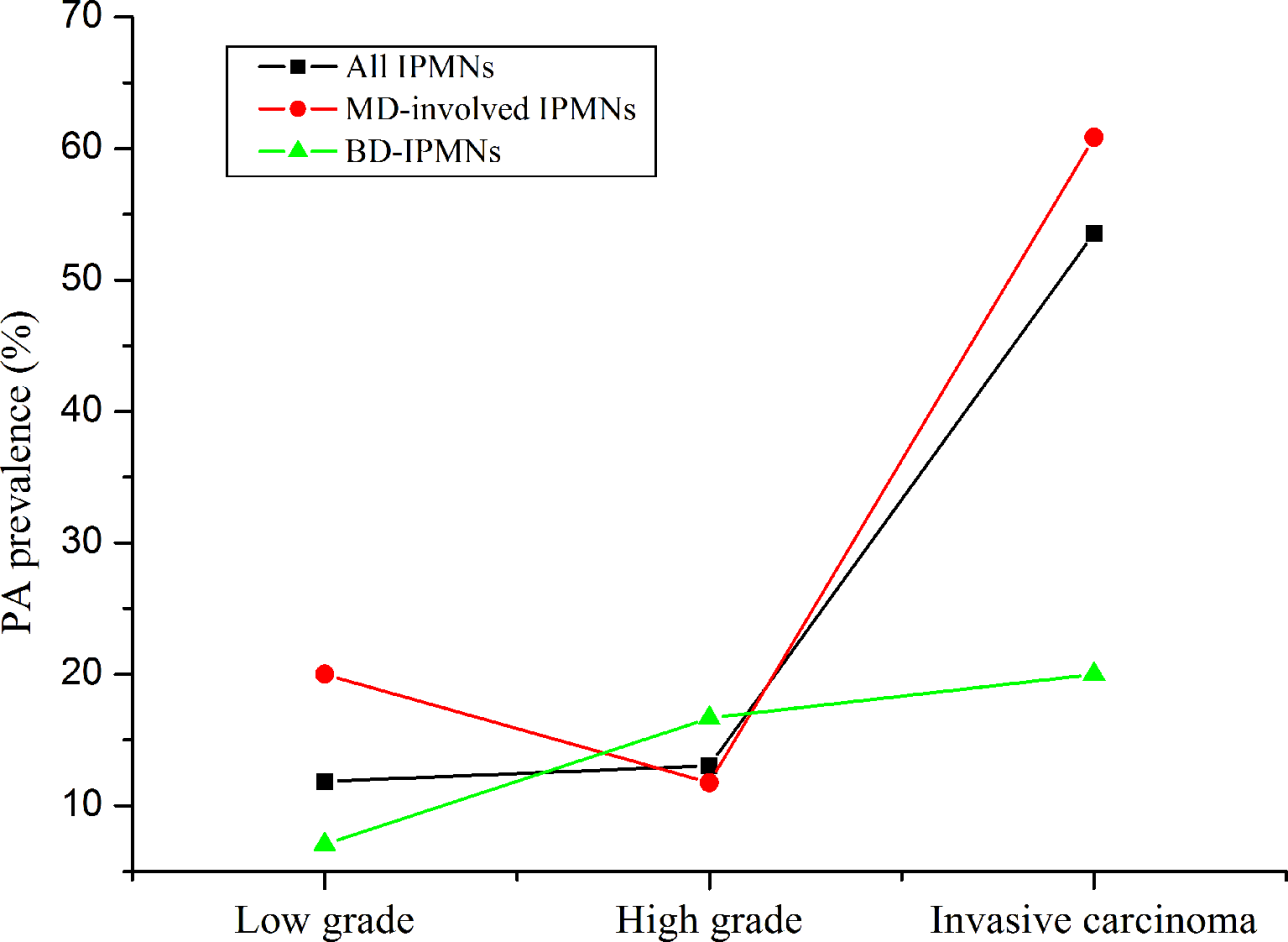
The prevalence of pancreatic atrophy in all intraductal papillary mucinous neoplasms (IPMNs), MD-involved IPMN, and BD-IPMNs. The p-values for trends were less than 0.01 and was 0.21. “Low” means low and intermediate grade.

**Figure 2:**
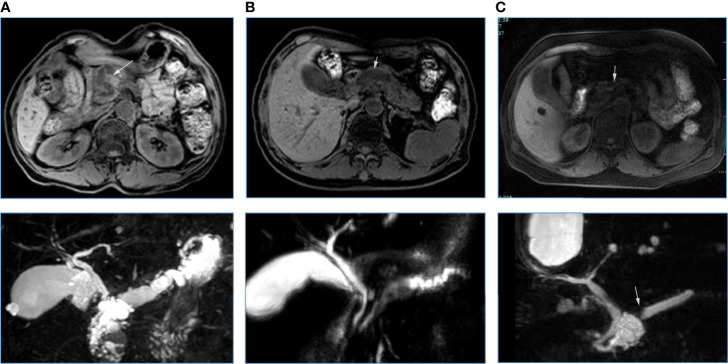
Three cases of IPMNs with **(A, B)** or without **(C)** pancreatic atrophy. A: A 67-year-old woman with IPMN-derived invasive carcinoma in pancreatic head (white arrow). Atrophy occurred in the pancreatic body and tail. Pancreatic duct dilatation occurred (below). B: A 61-year old man with low-grade IPMN in the pancreatic head. Pancreatic duct dilatation occurred (white arrow), but pancreatic atrophy was not observed. C: A 57-year old woman with low–moderate grade of IPMN in pancreatic head. An abrupt change in the caliber of the main pancreatic duct (bottom white arrow) with body pancreatic atrophy was observed (the ratio between the main pancreatic duct diameter and the width of the total gland was 0.54, white arrow).

The occurrences of malignancy and invasive carcinoma in patients with PA were all higher than those without PA (52.9% vs. 22.4%; 44.2% vs. 8.6%) (both P < 0.01). Subsequently, we assessed the correlation between the prevalence of PA and grade of IPMNs (**Figure 1**). The prevalence of PA in all IPMNs and MD-involved IPMNs were increased with tumor the grade (p < 0.01). Such trends were also observed in BD-IPMNs, but no statistical significance was found (P = 0.21). Three cases of IPMNs with or without PA are shown in **Figure 2**.

### The Association Between PA and Malignant IPMNs

Next, we observed the association between PA and malignancy or invasive carcinoma in IPMNs (**Tables 2**, **3**). Both univariate and multivariate logistic regression models showed that the occurrence of PA was an independent risk factor for malignancy (OR = 7.78, 95% CI: 2.62-23.10) or invasive carcinoma (OR = 2.69, 95% CI: 1.07-6.78) in IPMNs after being adjusted with confounders such as the tumor size, presence of MN, and MPD dilatation. The occurrence of the cyst size ≥3.0 cm and MPD diameter ≥1.0 cm were significantly associated with a higher risk of malignancy in IPMNs but not with invasive carcinoma.

**Table 2:** Associated factors with malignancy in IPMNs.

Variables	Model 1	Model 2	Model 3
	OR (95% CI)	OR (95% CI)	OR (95% CI)
Age	0.97 (0.94-1.01)	0.97 (0.93-1.01)	0.97 (0.93-1.02)
Size (≥ 3.0 cm vs. < 3.0 cm)	2.55 (1.14-5.67)	2.76 (1.20-6.32)	2.67 (1.15-6.16)
Mural nodule (yes vs. no)	3.90 (1.44-10.59)	4.05 (1.46-11.26)	4.05 (1.46-11.25)
MPD (≥ 1.0 cm vs. < 1.0 cm)	3.45 (1.30-9.17)	3.35 (1.25-9.02)	3.31 (1.22-8.96)
PA (yes vs. no)	2.78 (1.10-7.00)	2.75 (1.20-6.32)	2.69 (1.07-6.78)

Model 2 was additionally adjusted for sex and tumor location.

Model 3 was further adjusted for diabetes.

MPD, main pancreatic duct; PA, pancreatic atrophy; OR, odds ratio.

**Table 3:** Associated factors with invasive carcinoma in IPMN patients.

Variables	Model 1	Model 2	Model 3
	OR (95% CI)	OR (95% CI)	OR (95% CI)
Age	0.97 (0.93-1.02)	0.98 (0.93-1.02)	0.98 (0.93-1.03)
Size (≥3.0 cm vs. <3.0 cm)	0.85 (0.30-2.40)	0.83 (0.28-2.48)	0.72 (0.24-2.21)
Mural nodule (yes vs. no)	4.63 (1.54-13.95)	4.56 (1.48-14.13)	4.87 (1.56-15.22)
MPD (≥ 1.0 cm vs. < 1.0 cm)	2.71 (0.85-8.67)	2.76 (0.86-8.93)	2.68 (0.82-8.80)
PA (yes vs. no)	7.85 (2.70-22.83)	7.92 (2.70-23.23)	7.78 (2.62-23.10)

Model 2 was additionally adjusted for sex and tumor location.

Model 3 was further adjusted for diabetes.

MPD, main pancreatic duct; PA, pancreatic atrophy; CI, confidence interval; OR, odds ratio.

### The Association Between PA and Invasive Carcinoma in MD-Involved IPMNs

We examined whether the correlation between PA and malignancy or invasive carcinoma was present in MD-involved IPMN patients (**Table 4**). Univariable analysis showed that PA were associated with invasive carcinoma (OR = 6.54, 95% CI: 2.00-21.38; OR = 7.13, 95% CI: 2.51-20.26), but not with malignant IPMNs. Multivariate analysis further demonstrated that PA were independently associated with invasive carcinoma (OR = 9.72, 95% CI: 2.43-38.88).

**Table 4:** Associated factors with malignancy or invasive carcinoma in MD-involved IPMNs.

	Variables	Univariate	Model 1	Model 2
			OR (95% CI)	OR (95% CI)
Malignant IPMNs	Age	0.99 (0.94-1.04)	0.98 (0.92-1.03)	0.99 (0.93-1.05)
	Size (≥3.0 cm vs. <3.0 cm)	2.14 (0.86-5.30)	1.94 (0.71-5.34)	2.15 (0.70-6.62)
	Mural node (yes vs. no)	4.13 (1.20-14.17)	4.16 (0.97-17.91)	3.31 (0.74-14.75)
	MPD (≥1.0 cm vs. <1.0 cm)	2.69 (1.06-6.85)	1.66 (0.58-4.70)	1.74 (0.57-5.12)
	PA (yes vs. no)	2.50 (0.98-6.37)	1.94 (0.66-5.67)	1.96 (0.65-5.93)
Invasive carcinoma	Age	0.98 (0.93-1.03)	0.98 (0.91-1.04)	0.98 (0.92-1.05)
	Size (≥3.0 cm vs. <3.0 cm)	1.18 (0.44-3.22)	0.65 (0.19-2.26)	0.43 (0.09-1.99)
	Mural node (yes vs. no)	6.54 (2.00-21.38)	4.41 (1.09-17.82)	4.02 (0.84-19.23)
	MPD (≥1.0 cm vs. <1.0 cm)	2.22 (0.82-5.98)	1.55 (0.45-5.37)	1.42 (0.35-5.67)
	PA (yes vs. no)	7.13 (2.51-20.26)	6.78 (2.01-22.84)	9.72 (2.43-38.88)

Model 2 was additionally adjusted for diabetes and tumor location.

Malignant IPMNs: high grade and invasive carcinoma.

MPD, main pancreatic duct; PA, pancreatic atrophy; CI, confidence interval; OR, odds ratio.

### ROC Curves in Identifying Malignancy or Invasive Carcinoma

**Figure 3:**
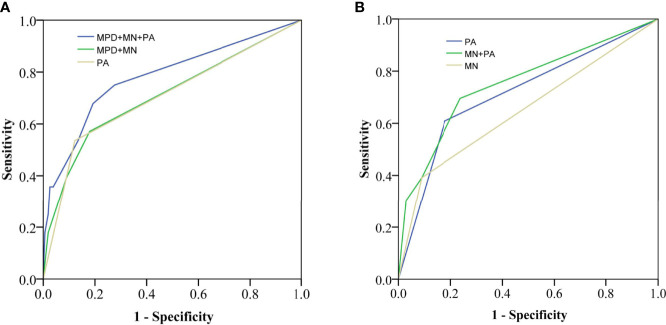
Receiver operating characteristic (ROC) curves for identifying invasive carcinoma in all IPMNs **(A)** [area under the curve (AUC) = 0.78 vs. 0.71, 0.71] and MD-involved IPMNs **(B)** (AUC = 0.76 vs. 0.65, 0.71). MPD, main pancreatic duct; MN, mural nodule; PA, pancreatic atrophy.

The ROC curves in identifying malignancy or invasive carcinoma are presented in **Figure 3**. PA had a similar performance with MPD plus MN (the AUC was both 0.71). The combined MPD diameter, MN, and PA had a higher performance (AUC = 0.78) than that of the MPD diameter plus MN or PA alone (P = 0.03) in identifying invasive carcinoma in all IPMNs.

PA also had acceptable performance in identifying invasive carcinoma in MD-involved IPMNs (AUC = 0.71), which was higher than that of MN (AUC = 0.65) (P = 0.02). Moreover, the AUC of MN plus PA (AUC = 0.76) was higher than that of MN (AUC = 0.65) or PA (AUC = 0.71) alone in MD-involved IPMNs.

## Discussion

An abrupt change in the caliber of MPD with distal PA has been regarded as a risk factor for malignant IPMNs or feature for surgical resection in IAP and ACG guidelines (3, 7). However, little is known about the association between PA alone and malignant IPMNs. In the present study, our data demonstrated that PA alone was an associated factor for malignant IPMNs or invasive carcinoma. PA was also associated with invasive carcinoma in MD-involved IPMNs. Moreover, PA alone, PA plus MN, and PA Plus MN and MPD all had acceptable performance in identifying malignant IPMNs and invasive carcinoma (AUC > 0.70).

The occurrence of PA in IPMNs is not uncommon. Two recent radiological studies showed that 23% of IPMN patients had PA in computed tomography (CT) images (16, 17), which was similar to the occurrence of the MPD size ≥1.0 cm. Our data showed that 18.2% of IPMN patients had PA that was consistent with those previous results. Moreover, a recent study reported that PA occurred in 30% IPMN patients (20). Therefore, the role of PA in IPMNs should draw people’s attention extensively.

Several guidelines have showed malignant features or surgical indications for IPMNs (2), such as MN or enhanced solid component, MPD > 1.0 cm, and tumor size ≥ 3.0 cm. The diagnostic performances of the 2017 revised International Consensus Guidelines have been validated by some studies (16, 17, 28). Lee et al. (16) indicated that the diagnostic performance of CT and MRI (based on high-risk stigmata and worrisome features) were both good, AUC = 0.83 for CT and AUC = 0.86 for MRI. Min et al. reported that the diagnostic accuracy of high-risk stigmata (MPD diameter ≥10 mm, MN ≥5 mm, and obstructive jaundice) was 73.7% for CT and 75.4% for MRI (n = 175). Several nomogram models based on clinical, high-risk stigmata and/or worrisome features also indicated an excellent discrimination performance (4, 29, 30). However, abrupt change in the caliber of MPD with distal PA or PA alone was not included in those models. Our data showed that the combined model of MPD plus MN had a similar discrimination performance (AUC = 0.71) to previous data (AUC = 0.69) (30). The addition of PA significantly improved the performance of the combined model of MPD plus MN in identifying malignant IPMNs (AUC = 0.78 vs. 0.71, P < 0.05). The performance of PA plus MPD and MN was comparable to those reported models (4, 29).

A single biomarker in isolation was also used to identify malignancy in IPMNs. Sugimoto et al. showed that the diagnostic performance of an MPD diameter cut-off of 7.2 mm for malignant neoplasms was acceptable (AUC = 0.70, 95% CI: 0.59-0.81). Kim et al. (31) demonstrated that the accuracy was 73.8% for elevated serum CA19-9, 73.3% for MPD >5 mm, and 77.7% for MN in identifying malignant IPMNs. A recent study indicates that mucin 5AC (MUC5AC) in a circulating extracellular vesicle can predict high-grade IPMNs with an AUC of 0.73 and predict invasive carcinoma with an AUC of 0.91 (32). Our previous study also reported that serum ferritin had acceptable performance (AUC = 0.67) (33). In the present study, our data further showed that the diagnostic power of PA alone was comparable to that of MPD or MN (AUC = 0.71) and the serum biomarkers of CA19-9 or serum ferritin or a circulating extracellular vesicle in identifying malignant IPMNs but lower than that of the circulating extracellular vesicle in identifying invasive carcinoma (32).

CT, MRI, and endoscopic ultrasonography (EUS) are all useful for IPMN diagnosis or malignancy identification. A recent meta-analysis showed that contrast-enhanced EUS had good performance (accuracy of 89.6%, sensitivity of 88.2%, and specificity of 79.1%) in identifying a mural nodule (34). EUS also has a great role in preoperative biopsy. Crinò et al. reported a new EUS-guided cyst-wall biopsy in pancreatic cysts (35), which can be used for pathological examinations. Moreover, EUS-guided confocal laser endomicroscopy has great potential in identifying high-grade dysplasia/adenocarcinoma with high sensitivity and accuracy (36, 37). However, we did not observe the role of EUS for PA evaluation because EUS is not routinely performed for IPMNs in China. Because MRI or MRCP may have an advantage in differentiating the IPMN type and some CT examinations of our patients were performed outside, we only included those patients with MRI examinations.

Our study has several limitations. First, only the patients who underwent surgery and MRI examinations were included, which may cause selection bias. The generalization of our results should be confirmed by other studies, even though pathological studies have shown that PA was related to invasive carcinoma or malignant IPMNs (8, 11). Second, PA was evaluated by radiological examinations, not by pathological examinations. Third, some worrisome features, such as elevated CA19-9 levels and thickened enhancing cyst walls, were not considered as confounders because of the small sample size of IPMNs with PA. Finally, we did not analyze the association between PA and malignancy in BD-IPMNs because the incidence of PA was low in BD-IPMNs.

In conclusion, the occurrence of PA in IPMNs was common. PA was associated with malignant IPMNs and invasive carcinoma. PA alone or combined with MPD and MN had an acceptable performance in predicting malignancy in IPMNs. Our data support that PA could be regarded as one of the associated factors or worrisome features in the guidelines.

## Data Availability Statement

The original contributions presented in the study are included in the article/supplementary material. Further inquiries can be directed to the corresponding authors.

## Ethics Statement

The retrospective study was approved by the Ethic Committee of Affiliated Hospital of Nanjing University of Chinese Medicine. Written informed consent for participation was not required for this study in accordance with the national legislation and the institutional requirements.

## Author Contributions

CW and XiaoC participated in the design of the study. TL, XinC and XiaoC wrote the manuscript. TL, XinC, JL, YC, and WC collected and analyzed the data. TL, XinC, CW and ZW contributed to interpretation of data and preparation of the manuscript. All authors read and approved the final manuscript.

## Funding

Peak academic talent training fund of Jiangsu Province Hospital of Chinese Medicine(y2018rc04); Science and Technology Development Plan fund of Chinese Medicine of Jiangsu Province (ZD201907) and National Natural Science Foundation of China (No. 81773460).

## Conflict of Interest

The authors declare that the research was conducted in the absence of any commercial or financial relationships that could be construed as a potential conflict of interest.

## Publisher’s Note

All claims expressed in this article are solely those of the authors and do not necessarily represent those of their affiliated organizations, or those of the publisher, the editors and the reviewers. Any product that may be evaluated in this article, or claim that may be made by its manufacturer, is not guaranteed or endorsed by the publisher.
